# Spatiotemporal control of CRISPR/Cas9 gene editing

**DOI:** 10.1038/s41392-021-00645-w

**Published:** 2021-06-20

**Authors:** Chenya Zhuo, Jiabin Zhang, Jung-Hwan Lee, Ju Jiao, Du Cheng, Li Liu, Hae-Won Kim, Yu Tao, Mingqiang Li

**Affiliations:** 1 Laboratory of Biomaterials and Translational Medicine, Center for Nanomedicine, The Third Affiliated Hospital, Sun Yat-sen University, Guangzhou, China; 2 Institute of Tissue Regeneration Engineering (ITREN), Dankook University, Cheonan, South Korea; 3 Department of Nuclear Medicine, The Third Affiliated Hospital, Sun Yat-sen University, Guangzhou, China; 4 PCFM Lab of Ministry of Education, School of Materials Science and Engineering, Sun Yat-sen University, Guangzhou, China; 5 Department of Gynecology and Obstetrics, The Third Affiliated Hospital, Sun Yat-sen University, Guangzhou, China; 6 Guangdong Provincial Key Laboratory of Liver Disease Research, Guangzhou, China

**Keywords:** Gene therapy, Biomaterials

## Abstract

The clustered regularly interspaced short palindromic repeats (CRISPR)/associated protein 9 (CRISPR/Cas9) gene editing technology, as a revolutionary breakthrough in genetic engineering, offers a promising platform to improve the treatment of various genetic and infectious diseases because of its simple design and powerful ability to edit different loci simultaneously. However, failure to conduct precise gene editing in specific tissues or cells within a certain time may result in undesirable consequences, such as serious off-target effects, representing a critical challenge for the clinical translation of the technology. Recently, some emerging strategies using genetic regulation, chemical and physical strategies to regulate the activity of CRISPR/Cas9 have shown promising results in the improvement of spatiotemporal controllability. Herein, in this review, we first summarize the latest progress of these advanced strategies involving cell-specific promoters, small-molecule activation and inhibition, bioresponsive delivery carriers, and optical/thermal/ultrasonic/magnetic activation. Next, we highlight the advantages and disadvantages of various strategies and discuss their obstacles and limitations in clinical translation. Finally, we propose viewpoints on directions that can be explored to further improve the spatiotemporal operability of CRISPR/Cas9.

## Introduction

The emergence of the CRISPR gene editing system has revolutionized genetic engineering.^1,2^ There are two major categories of CRISPR/Cas systems, which are branched into six types (types I to VI).^3,4^ Among them, type I (Cas3), II (Cas9), IV (Csf1), and V (Cas12) target DNA, while type III (Cmr3) and VI (Cas13) are considered to target RNA specifically.^5^ The most studied CRISPR/Cas system is the type II CRISPR/Cas9 system, which serves as an adaptive immune system and implements defense against phage infection in bacteria and archaea. The Cas9 protease specifically recognizes the sequence with a protospacer adjacent motif (PAM) under the guidance of two noncoding RNAs (CRISPR RNA (crRNA) and trans-activating crRNA (tacrRNA)), which can also be engineered to be a single guide RNA (sgRNA).^3^ Once the target DNA is recognized, its double strands are sundered by two domains of Cas9 nuclease (HNH domain and RuvC domain). The broken double-stranded DNA can be corrected through nonhomologous end-joining (NHEJ) or homology-directed repair (HDR), resulting in gene insertions/deletions (indels) and gene mutations.^6,7^ Gene frameshift mutations and premature introduction of stop codons caused by NHEJ may lead to gene knockout and premature inhibition of gene expression, respectively. HDR requires donor DNA templates, which can result in the desired gene editing.

Because of the ease of design and capability of editing multiple sites simultaneously in the genome, the CRISPR/Cas9 system holds vast potential in treating various genetic and infectious diseases,^8^ such as nonmonogenic cardiovascular diseases, monogenic cataract diseases, cancer, metabolic disorders, human immunodeficiency virus (HIV) infection and Alzheimer’s disease.^3,4,9–13^ Although these exciting possibilities exist, CRISPR/Cas9 gene editing still requires more precise control over time and spatial dimensions in complex biological systems.^8,14,15^ More specifically, when CRISPR/Cas9 gene editing is used, gene perturbations should be avoided at certain stages of cell differentiation or tissue development. Therefore, CRISPR/Cas9 gene editing must be localized to specific cells or tissues at a specific time.^16^ Additionally, the off-target effects and genotoxicity will increase with the increasing Cas9 activity^8,14,17^ and are one of the most important issues in the process of CRISPR gene therapy.^18^ Thus, to minimize genotoxicity and off-target activity and maximize therapeutic efficacy, spatiotemporal control of CRISPR/Cas9 activity must be achieved in complex biological tissues.^19–21^ Additionally, the technology of the precise spatiotemporal control of CRISPR/Cas9 activity will be beneficial in basic research and translational applications.^15,22^

**Fig. 1:**
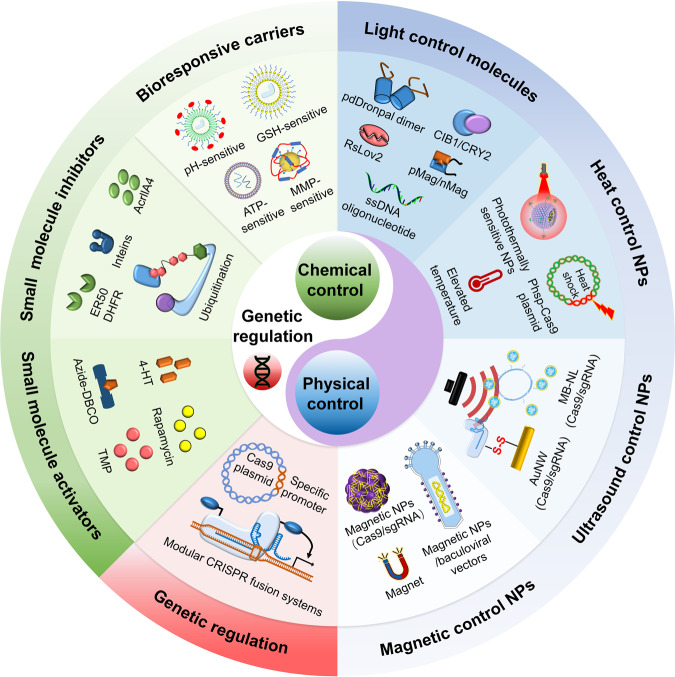
Schematic illustration shows various strategies for spatiotemporal control of CRISPR/Cas9 gene editing

To date, the spatiotemporal control of CRISPR/Cas9 gene editing remains a daunting challenge for the robust effectuation of gene editing in clinical applications.^20,23^ For this situation, genetic regulation, chemical, and physical strategies have been studied to strengthen the conditional regulation of CRISPR/Cas9 function (Fig. 1), such as small molecule activation, small molecule inhibition, cell-specific promoters, bioresponsive delivery carriers and optical/thermal/ultrasonic/magnetic activation of the CRISPR/Cas9 system.^24–35^ These strategies are suboptimal, and each has its shortcomings. For example, most small molecules used for chemical activation (particularly rapamycin and doxycycline) may induce potential cytotoxicity toward both targeted and nontargeted cells and may even lack high-level spatial specificity and reversibility, making them difficult to explore for in vivo studies.^18^ Additionally, one of the current problems with photoactivation is that light signals have difficulty penetrating deep tissues in in vivo applications. The strong absorption and scattering of light by turbid and complex biological tissues is the main reason for this problem.^9^ The goal of these gene editing technologies is to be safely and effectively applied in vivo and clinically. However, most of the strategies are only studied using cell lines in vitro, while their suitability for in vivo programmable applications remains unclear. Thus, comparative analyses among different methods to precise control of the spatiotemporal CRISPR/Cas9 gene editing activity are needed.

This review discusses the state-of-the-art genetic regulation, chemical and physical strategies for the spatiotemporal control of CRISPR/Cas9 activity, from the underlying mechanism and rational design to the resultant controllability and gene editing characteristics, and provides the in-depth insight into their challenges and opportunities.

## Genetic regulation

CRISPR/Cas9 technology provides a diversified sequence-specific gene regulation “tool kit” by using catalytically impaired Cas9 (dCas9), which does not cleave DNA but can precisely and specifically bind DNA under the guidance of specific sgRNA.^36^ Therefore, dCas9 can be designed to recruit different transcription activators or repressors for targeted gene activation or repression (Table 1). Rational engineering of the sgRNA molecule by the fusion of aptamers can also regulate target gene transcription. dCas9 can also be designed to recruit epigenetic modifiers to achieve epigenetic modifications at specific genomic locations. Controlling the editing of target genes through cell-specific promoters is another gene regulation strategy with stronger specificity and low off-target effects (Table 1).^37^

**Table 1:** Examples of genetic regulation strategies of CRISPR gene editing

Control type	Cell or organism models	Edited gene	Key molecule / structure	Ref.
Modular CRISPR fusion system	MSC, HEK-293T, hPSC; yeast cell, HeLa cell	adipogenic genes, human *ASCL1, IL1RN, ZFP42* and *OCT4* genes, pluripotency gene *NANOG*, *GFP* reporter gene	VP64, p65, copies of VP16, VP64-p65-Rta tripartite, multiple sgRNAs, AcrIIA1-4, KRAB, SID4X	^31,32,38,39,41–45,48,60^
Cell-specific promoter	Huh7 cell, HEK-293T cell, HepG2.2.15 cell, mouse T cell, B cell, neutrophil, monocyte, macrophage, and spleen cell, zebrafish, *C. elegan*s	hepatitis B virus (HBV) genome, mouse *CD2* gene, macrophage-specific gene *sgNtn 1*, zebrafish *urod* gene, *C. elegans* somatic cell *DPY-5*, *LON-2*, and *GFP* gene	liver-specific promoter, macrophage-specific promoter, CD4 promoter, erythrocyte-specific gata1 promoter, Egg cell-specific promoter	^37,52–55,57^

*MSC* mesenchymal stem cell, *HEK-293T cell* human embryonic kidney 293T cell, *hPSC* human pluripotent stem cell, *Huh7 cell* human liver cancer cell line, *GFP* green fluorescent protein, *VP64, p65, VP16 and Rta* transcription activation domains, *KRAB, SID4X, MXl1 and WRPW* transcription repression domain

### Modular CRISPR fusion systems

#### Transcription activation

**Fig. 2:**
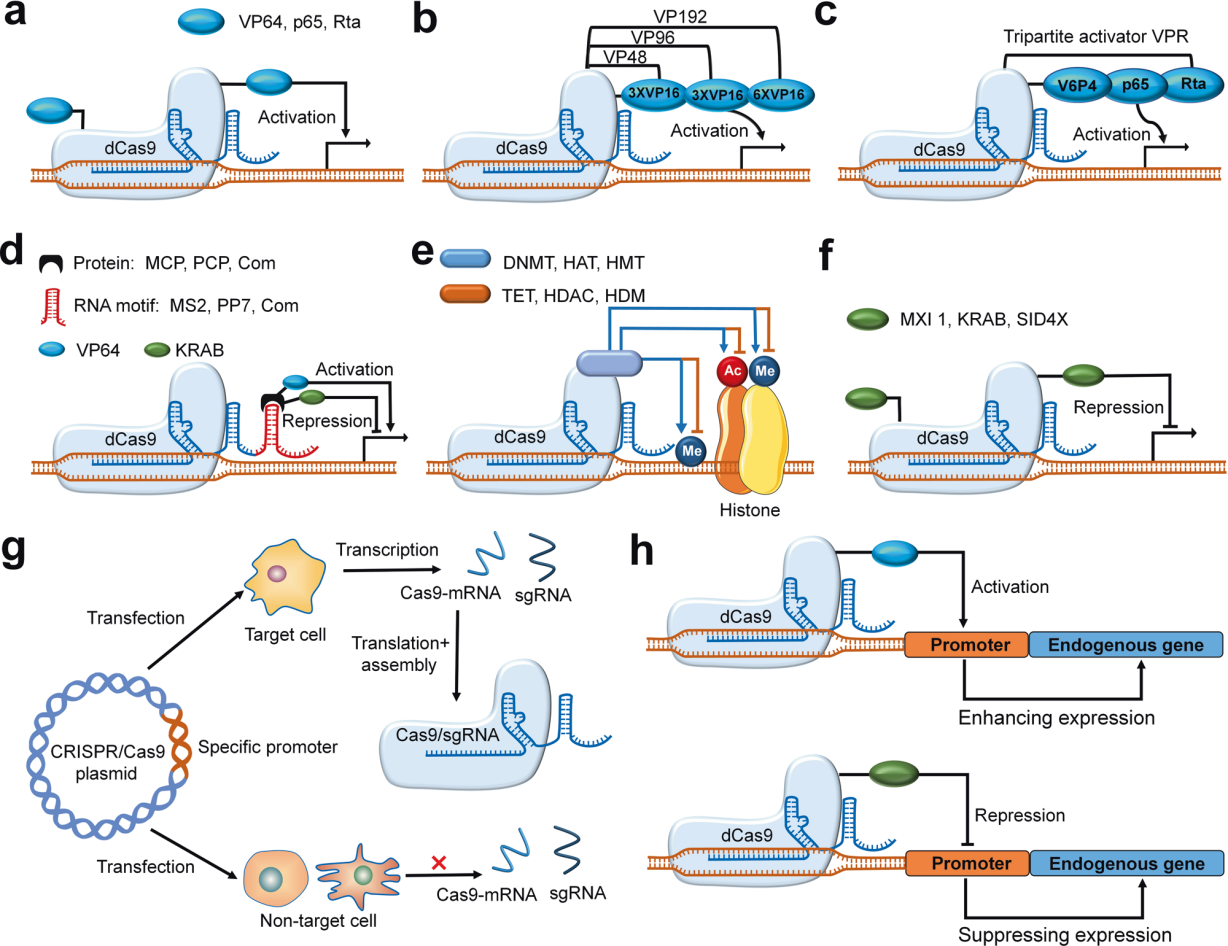
The strategies of genetic regulation. Modular CRISPR fusion systems for efficient transcription repression and activation (**a–f**): **a** dCas9 can be used to mediate transcriptional activation of target genes when fused to small-molecule activation domains such as Rta, p65, and VP64. **b**, **c** The methods for enhanced transcription activation through fusing multiple copies of VP16 or a tripartite activator domain termed as VPR (a fusion of VP64, p65AD, and Rta) to dCas9. **d** RNA aptamer (MS2, PP7 or Com)-based recruitments of cognate RNA binding proteins (MCP, PCP, or Com) for engineering of sgRNA. **e** Epigenetic regulation at targeted site through the interaction between dCas9 and enzyme domains (TET/DNMT, HDM/HMT, or HDAC/HAT). **f** The methods for transcription repression through fusing different repressor domains (MXI1, KRAB, and SID4X) to dCas9. Cell-specific promoter for the control of Cas9 activity (**g**, **h**): **g** The CRISPR/Cas9 plasmid driven by the specific promoter, resulting the transcription of Cas9-mRNA and sgRNA in target cell, but not non-target cell. **h** A dCas9-activator/inhibitor fusion, which specifically targets the promoter or enhancer region of endogenous genes, induces the up/down-regulation of some endogenous loci

The basic principle of this method is that dCas9 cannot cut DNA because of a mutation in its two domains (RuvC and HNH nuclease domains), but it can target genomic loci with the guidance of sgRNA.^3^ Thus, when bound to small-molecule activation domains such as Rta, p65AD and VP64, dCas9 with a specific sgRNA can mediate basic transcriptional activation of the target gene (Fig. 2a).^38–40^ For example, Gilbert et al. and Mail et al. fused the activation domain p65 or VP64 to dCas9 to form the fusion protein dCas9-VP64 or dCas9-p65, respectively.^38,41^ These fusion proteins and sgRNA targeting *Gal4* upstream activation sequence (UAS) were simultaneously transfected into HEK-293T cells, which expressed GFP proteins. Compared with cells expressing dCas9 alone, HEK-293T cells transfected with the dCas9-VP64 fusion protein or dCas9-p65 fusion protein showed a 25-fold or 12-fold increase in GFP fluorescence intensity, respectively.^38^ In addition to dCas9 engineering, sgRNA engineering can also regulate the activity of the CRISPR/Cas9 system by binding to protein-interacting RNA aptamers to recruit different transcription activation domains, such as VP64 or p65, to activate gene expression. Zalatan et al. observed significant reporter gene expression driven by scRNA constructs with different RNA hairpin structures, such as MS2, PP7, or Com RNA hairpins. These RNA hairpins can recruit their protein-interacting RNA aptamers (MCP, PCP, Com), which are connected to the VP64 activation domain, resulting in the promotion of target gene expression (Fig. 2d).^42^

#### Enhanced transcription activation

The transcription activation efficiency can be further improved using different optimization strategies, including the addition of multiple copies of activation domain VP16 (such as dCas9-VP48, -VP96, -VP192) (Fig. 2b), the design of a tripartite activator domain VPR (Fig. 2c) or the insertion of multiple sgRNAs. For example, Balboa et al. reported that the fusion of dCas9 with 12 repeats of VP16 (dCas9-VP192) effectively activated and increased target gene expression levels by 70-fold.^43^ Guo et al. and Chavez et al. used dCas9-VP64 as a scaffold connected to p65 and Rta, resulting in the formation of a hybrid dCas9-VP64-p65-Rta tripartite activator (termed dCas9-VPR) (Fig. 2c).^44,45^ As expected, the three-part fusions promoted transcriptional activity. The strategy of multiple sgRNAs to improve the activation efficiency was studied by Maeder et al.^39^ Sixteen prevalidated sgRNAs were designed to target sequences in three DNase I hypersensitivity sites, locating upstream, downstream, or at the start site of *VEGFA* gene transcription. The VEGFA protein expression levels induced by dCas-VP64 with multiple sgRNAs (V1, V2, V3, and V4) were significantly greater than the expected additive effects of individual activators.^39^

#### Transcription repression

The realization of this method is based on the mechanism: the dCas9/sgRNA complex produces sequence-specific gene suppression by blocking RNA polymerase to interfere with transcription elongation.^36,46^ The transcription repression efficiency can be improved by fusing small-molecule transcription repression domains, such as KRAB, SID4X, and MXl1, to dCas9/sgRNA (Fig. 2f).^38,42,47–50^ Interestingly, the inhibition efficiency of transcriptional repressors binding to the amino terminus of dCas9 is higher than that of transcriptional repressors binding to the carboxyl terminus of dCas9.^47^ For example, Gilbert et al. showed that coupling dCas9 to various inhibitory chromatin modifier domains, such as the KRAB domain of Kox1, CS domain of HP1, and WRPW domain of Hes1, robustly inhibited *GFP* reporter gene expression under the guidance of an sgRNA targeting the *GFP* gene in HEK-293T-GFP reporter cells.^38^ Additionally, compared with cells expressing dCas9, cells expressing the dCas9-KRAB fusion protein showed a 5-fold reduction in the GFP signal and those with dCas9-CS or dCas9-WRPW fusion protein showed a 2-fold reduction in the GFP signal.^38^ Fusing small-molecule transcription repression domains to sgRNA is another choice to inhibit gene expression. Zalatan et al. designed sgRNA constructs by fusing the repression domain Com-KRAB to the RNA binding domain Com to target the transcriptional start site (Fig. 2d), causing significant repression of GFP compared with dCas9 alone.^42^

#### Epigenetic modification

Epigenetic-modifying enzymes, including histone demethylase (HDM)/methyltransferase (HMT), histone acetyltransferase (HAT)/deacetylase (HDAC), and DNA-demethylating enzymes (TET)/methyltransferase (DNMT), are also applied to fuse with dCas9 to expand its functionality and control diverse epigenetic states of targeted endogenous genes (Fig. 2e).^51^

### Cell-specific promoter

#### Construction of cell-specific promoters

The promoter is an essential element that controls the expression of a gene cassette. Thus, constructing a cell-specific promoter to drive the expression of Cas9 nucleases in the target cell is a direct and effective way to realize the spatiotemporal control of CRISPR gene editing and counteract undesired side effects in nontarget cells (Fig. 2g).^37,52^ Recently, some scientists have successfully established the CRISPR/Cas9 system with organ-specific promoters that can pointedly drive gene editing in macrophages, *Caenorhabditis elegans*, monocytes, zebrafish, and liver cells.^37,52–56^ For example, the original chicken β-actin promoter of the Cas9 expression plasmid, pX330, was replaced with a macrophage-specific promoter (CD68 promoter) to obtain the Cas9 macrophage-specific expression plasmid pM330 (CD68-Cas9 plasmid).^37^ A sgRNA targeting *Ntn1* (sgNtn1) was then encoded into the plasmid pM330, followed by the encapsulation of a specific pM330/sgNtn1 plasmid into cationic lipid-assisted PEG-*b*-PLGA nanoparticles (CLAN) to form a CLANpM330/sgNtn1 platform.^37^ Driven by the CD68 promoter and guided by sgNtn1, the CLANpM330/sgNtn1 platform specifically expressed the Cas9 protein and successfully caused *Ntn1* knockout in monocytes and their precursor monocytes in vivo and in vitro but not in other types of cells.^37^ A similar study by Wang et al. found that, in *Arabidopsis*, an unmodified CRISPR/Cas9 system cannot function in egg cells and one-cell-stage embryos. They speculated that this might be caused by the weak activity of the constitutive cauliflower mosaic virus 35S promoter (CaMV 35 S) of the CRISPR/Cas9 system in egg cells.^57^ Therefore, they replaced the promoter of CaMV 35S with the egg cell-specific promoter EC1.2p to drive Cas9 expression in egg cells, creating homozygous or biallelic mutants for multiple target genes in *Arabidopsis* in the T1 generation.^57^ Moreover, compared with the single promoter, the integration of multiple egg cell-specific promoters significantly improved the efficiency of gene editing in the T1 generation.^57^

#### Specific activation of the promoter sequence

A dCas9-activator fusion that targets the promoter or enhancer region can also cause the upregulation of the target gene (Fig. 2h),^24,58^ representing another strategy to conduct spatiotemporal control of CRISPR/Cas9 gene editing. Hilto et al. constructed a programmable CRISPR/Cas9-based acetyltransferase by fusing the dCas9 protein to the catalytic core of the human acetyltransferase p300, which could catalyse the acetylation of histone H3 lysine 27 at its target site, resulting in robust expression of the target gene by activating promoters and enhancers.^59^ In a similar study, Perez-Pinera et al. showed that the expression plasmids of sgRNA targeting the *IL1RN* promoter sequence were co-transfected with the dCas9-VP64 expression plasmid into HEK-293T cells, causing synergistic and strong activation of human *IL1RN* gene expression.^60^ Because the magnitude of transcriptional activation achieved by one sgRNA generally ranges from low to ineffective, multiple sgRNAs are adopted to target the desired promoter region and promote stronger transcriptional activation.^58^ Cheng et al. reported that under the guidance of 3–4 sgRNAs targeting *IL1RN* promoters, dCas9-VP48 or dCas9-VP160 enhanced the activation of the endogenous *IL1RN* gene in HEK-293T cells.^61^ In summary, strategies, such as cell-specific promoter-controlled CRISPR/Cas9 expression technology and dCas9 activator-based activation of the promoter or enhancer region of target genes, provide other promising approaches to spatiotemporally control CRISPR/Cas9 gene editing in target cells or tissues.

### Comparison of different genetic regulation strategies

The mechanism of using modular CRISPR fusion systems to control the transcriptional repression and activation of target genes is that the functionality of dCas9 is expanded by fusing transcription activator or repressor modules. The fusion protein dCas9-effectors then recognize the desired genomic site via sgRNA guidance to affect the transcription of a gene of interest. However, not every combination of effector modules is active, and the magnitude of regulatory activity depends on which and how many effectors are fused to the recruitment module and varies in the tissues of different species. Compared with modular CRISPR fusion systems, cell-specific promoters can achieve better spatial specificity of the CRISPR/Cas9 gene editing system and reduce off-target effects.^37,52^ However, screening promoters with cell specificity and high activity is the key challenge of this method.

## Chemical control

The chemical strategies used to improve the spatiotemporal specificity of CRISPR/Cas9-mediated gene editing mainly include (1) the regulation of Cas9 nuclease activity through the fusion of dCas9 or normal Cas9 to small-molecule-triggered Cas9 binding and self-splicing inteins; (2) the inhibition of Cas9 nuclease activity by anti-CRISPR proteins or degrons; and (3) the design of bioresponsive delivery carriers to control the release of the CRISPR/Cas9 system in specific tissues or cells (Table 2).^30–32,62^

**Table 2:** Examples of chemical spatiotemporal control of CRISPR gene editing

Control type	Cell or organism models	Edited gene	Key molecule / structure	Ref.
Small molecule activators	HEK-293T cell, mouse zygote, STF3A cell line, HEK293-GFP cell, N2A cell	*GFP* reporter gene, *SOX2* gene, *EMX1, PPP1R12C, VEGFA, ASCL*	4-hydroxytamoxifen (4-HT), trimethoprim (TMP), rapamycin, azido-modified Cas9	^27,63,66,67^
Small molecule inhibitors	hPSC, HeLa cell, HEK-293T cell, K562 cell, *E. coli*, U2OS cell, NIH/3T3 cell	*CD71* and *CXCR4* gene, *E. coli* genome, endogenous *IL1RN* or *NANOG* gene, *VEGFA*, *GFP* or *BFP* reporter gene	AcrIIA1-4, ubiquitin ligase, unstable protein domains, DHFR and ER50	^17,26,69,70,85^
Bioresponsive delivery carrier	HEK-293 T cell, mouse hepatocytes, mouse liver, lung and spleen tissues	*GFP* reporter gene, mouse serum *PCSK9* gene	bioreducible BAMEA-O16B lipid NP, lipid molecules with different charges	^114,126^

*HEK-293T cell* human embryonic kidney 293T cell, *STF3A* a Cell that carries a Wnt-responsive luciferase reporter and also strongly expresses a Wnt ligand, *N2A cell* mouse neuroblastoma N2a cells, *K562 cell* human K562 erythroleukemia cell, *NIH/3T3* mouse embryonic cells, *hPSC* human pluripotent stem cell, *GFP* green fluorescent protein, *CD71* and *CXCR4* cell surface transmembrane proteins gene, *AcrIIA1-4* anti-CRISPR associated protein

### Small-molecule activators

#### Conformational changes

**Fig. 3:**
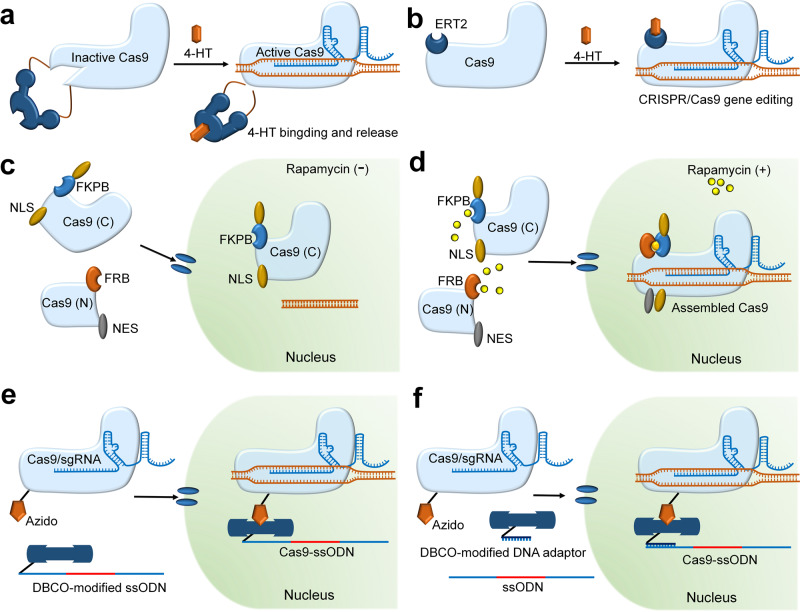
The strategies for combining small molecule activator to control the activity of Cas9. **a** Recovery of intein-inactivated Cas9 via 4-HT binding. **b** The fusion of Cas9 and the estrogen receptor (ERT2) is sequestered in the cytoplasm, but this fusion can enter the cell nucleus via the addition of 4-HT and form a Cas9/sgRNA complex. **c** Without the rapamycin, the Cas9(N)-FRB-NES fragment is accumulated in the cytoplasm, while the Cas9(C)-FKBP-NLS fragment is actively introduced into the cell nucleus. **d** In the presence of rapamycin, Cas9(N)-FRB-NES combines with Cas9(C)-FKBP-NLS, subsequently guided into the cell nucleus by NLS. The diagram of Cas9-ssODN conjugate formation and the principle of improving HDR efficiency (**e**, **f**): **e** Azido-modified Cas9 binds to DBCO-modified ssODN or **f** DBCO-modified DNA adapter and ssODN to form Cas9-ssODN conjugates, which effectively increases the local concentration of donor ssODN near the target area

The main mechanism of this method involves conformational changes in the Cas9 protein that can affect protein activity. Different functional small molecules, such as 4-hydroxytamoxifen (4-HT) and rapamycin, can control the conformational changes of proteins. Therefore, spatiotemporal control of Cas9 protein activity can be achieved through conformational changes in Cas9 controlled by adding small molecules. When a 412-amino acid intein is inserted into two different positions of the Cas9 protein (Ser219 or Cys574), inactivation of the Cas9 nuclease occurs; however, by adding 4-HT, the intein can be removed via conformational change and a self-cleaving reaction, resulting in reactivation of the Cas9 protein (Fig. 3a).^27^ The efficiency of protein activation varies from 3- to 10-fold depending on the insertion site of intein, and the ratio of on/off-target effects using this strategy can increase up to 25-fold compared with wild-type Cas9.^27^ Another study used 4-HT in human cells to turn on or off the activity of Cas9 variants.^63^ The combination of the Cas9 variant and hormone-binding domain of the estrogen receptor (ERT2) causes Cas9 to be sequestered into the cytoplasm (Fig. 3b). However, this fusion can enter the nucleus via the addition of 4-HT and form a Cas9/sgRNA complex to conduct gene editing (Fig. 3c).^63^ Through various optimizations, the final Cas9 variant (iCas) has higher editing capability at numerous sites with the addition of 4-HT but lower endonuclease activity in the absence of 4-HT.^63^ Therefore, temporal control over CRISPR/Cas9 gene editing can be conducted by adding 4-HT, which shows good results in improving gene-specific editing and reducing off-target effects.

Another mechanism using small molecules to control the conformational change of Cas9 is based on the chemically induced dimerization of split fragments of Cas9 protein.^64,65^ Zetsche et al. engineered a split Cas9 protein that was generated at two different split sites (Arg535 and Glu573) and produced C- and N-terminal Cas9 fragments that were bound with FK506 binding protein 12 (FKBP) and the FKBP rapamycin binding domain (FRB), respectively (Fig. 3c).^66^ The conditional reconstitution and activation of split-Cas9 was achieved via rapamycin-induced heterodimerization (Fig. 3d).^66^ Furthermore, to spatially separate the two fragments into different cellular compartments and prevent them from spontaneous reconstitution, a nuclear localization signal (NLS) and a nuclear export signal (NES) were attached to the C- and N-terminal Cas9 fragments, respectively, leading to decreased basal activity of the cas9 protein in the absence of rapamycin (Fig. 3c, d).

#### Oligonucleotide conjugates

Recent studies have also attempted to use site-specific Cas9-oligonucleotide conjugates to improve the accuracy and efficiency of gene editing.^67^ Accurate gene editing by the CRISPR/Cas9 system usually depends on HDR and requires sufficient donor DNA templates (preferably single-stranded oligodeoxynucleotides (ssODNs)) at the Cas9 cleavage site, otherwise making the HDR less efficient. To overcome these issues, Ling et al. used azide-containing noncanonical amino acids to modify a Cas9 protein to obtain chemically modified Cas9 mutants (Fig. 3e, f).^67^ These variants can bind to dibenzylcyclooctyne (DBCO)-modified ssODN (Fig. 3f) or DBCO-modified DNA adapter (Fig. 3g), both of which can recruit ssODNs to the cleavage complex and improve HDR-mediated gene editing efficiency.^67^

In summary, the development of small-molecule activators plays an important role in the spatiotemporal control of CRISPR/Cas9 activity. However, there are still some challenges. For example: (1) most small molecules, particularly rapamycin and doxycycline, may induce potential gene cytotoxicity toward both targeted and nontargeted cells during the process of chemical activation, making it difficult to be explored for in vivo studies;^18^ (2) Cas9 activity regulated using small-molecule activators often has limited dynamic range; in some cases, dCas9 even shows significant background activity in the absence of small-molecule activators,^68,69^ thereby interfering with precise spatiotemporal control of CRISPR/Cas9 gene editing.

### Small-molecule inhibitors

#### Inhibition of Cas9 activity by anti-CRISPR protein

**Fig. 4:**
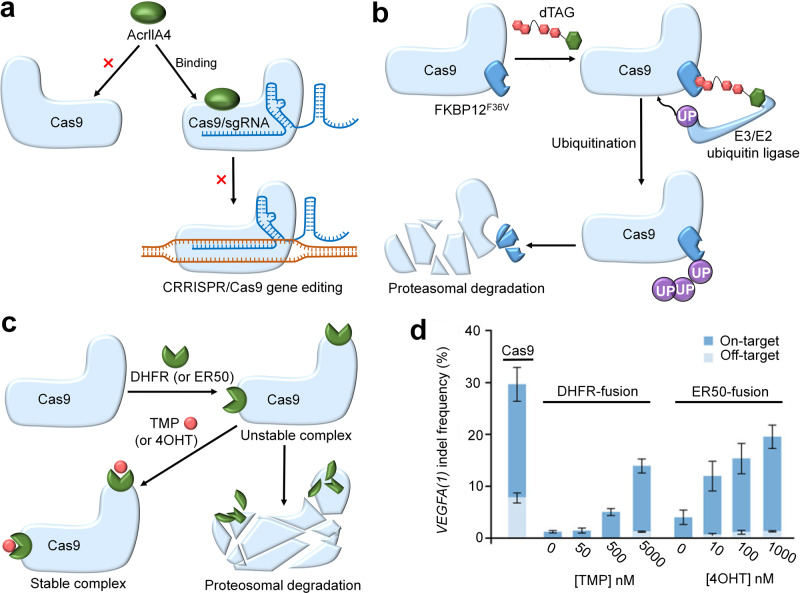
The strategies for combining small molecule inhibitors to control the activity of Cas9. **a** AcrIIA4 only binds to Cas9/sgRNA complexes, but not the Cas9 protein alone, and the resulting reassembled Cas9/sgRNA-AcrIIA4 complex cannot bind to target DNA. **b** Heterobifunctional dTAG molecules induce dimerization of FKBP12^F36V^ fusion chimeras and the E3/E2 ubiquitin ligase complex, leading to ubiquitination and proteasomal degradation of Cas9. **c** DHFR and ER50 are destabilized domains that can quickly target Cas9 protein for proteasomal degradation, but it can be stabilized upon addition of the corresponding small molecule TMP or 4OHT. **d** TMP and 4OHT-dose-dependent control of on and off-target activity of the complex Cas9-DHFR (ERR50) targeting *VEGFA* gene. Reproduced with permission.^26^ Copyright 2016, Springer Nature

Because elevated and lingering Cas9 activity often causes off-target effects, chromosomal translocations, and genotoxicity, Cas9 nuclease activity must be rapidly limited to a narrow time frame after target editing.^8^ Recently, some studies have shown that a class of small molecules, anti-CRISPR (Acr) proteins, are derived from a fierce coevolutionary arms race between bacteria and phages and can mediate deactivation of the CRISPR/Cas system.^70–73^ To date, the following Acr proteins have been identified: AcrIFs, AcrIIAs, AcrIICs, AcrVAs, AcrVIA, and AcrIIIB.^5,74^ Among them, AcrIFs were first found in the *P. aeruginosa* type I-F CRISPR/Cas system.^75^ AcrIIAs and AcrIICs show vast potential to inhibit Cas9 to regulate gene editing.^76^ AcrVAs can inhibit Cas12a which is another genome editing tool.^77^ AcrVIA can substantially attenuate RNA targeting and editing by Cas13a, which is the only member of the CRISPR-Cas systems that specifically targets and cleaves RNA.^5^ AcrIIIB can interfere with Cmr effector complexes to inhibit the type III-B CRISPR/Cas system.^78^ The discovery of Acr proteins makes it relatively easy to control Cas activity, thereby improving the spatial control of the CRISPR/Cas system. For example, AcrIIA4 is an anti-CRISPR protein that can only bind to the assembled Cas9/sgRNA complexes but not the Cas9 protein alone (Fig. 4a).^17^ The fusion of AcrIIA4 and Cas9/sgRNA leads to weaker binding of Cas9/sgRNA to the target DNA, thus hindering Cas9-mediated gene editing in human cells (Fig. 4a).^71^ It can be seen that, anti-CRISPR protein is a small molecule that can inhibit Cas9 protein activity. Thus, intracellular delivery of natural Cas9-specific anti-CRISPR proteins in the form of proteins or plasmids to serve as an on/off switch is an important mechanism to achieve the spatiotemporal control of CRISPR/Cas9 gene editing.

#### Degradation of Cas9 by small molecules

In many contexts, the timely degradation of Cas9 using small molecules may be better than the inhibition of Cas9 activity.^79,80^ Two strategies allow this goal. One strategy uses heterobifunctional molecules that colocalize the target protein and specific ubiquitin, resulting in engagement of the proteasomal degradation pathway; the other strategy directly fuses degrons to the target protein, causing target protein degradation.^81–84^ For example, Gangopadhyay et al. proposed a small-molecule degradation platform for Cas9 degradation (Fig. 4b).^8^ In this system, the Cas9 protein was linked to the FKBP12^F36V^ variant, which could connect with a specific E3/E2 ubiquitin ligase upon the addition of a heterobifunctional dTAG molecule, inducing ubiquitination and degradation of the fusion protein (Fig. 4b).^8,85^ Compared with ubiquitinated degradation, the operability of the direct fusion of degrons (DHFR or ER50) to Cas9 seems more convenient (Fig. 4c). DHFR (ER50) is a destabilized domain that can rapidly target the fusion protein for proteasome-mediated degradation but can be stabilized following the addition of small-molecule inhibitors trimethoprim (TMP) or 4OHT (Fig. 4c). Maji et al. observed an increased on-target to off-target ratio when *VEGFA* gene was edited by the Cas9-DHFR or Cas9-ER50 systems, which were treated with different doses of TMP (0–5000 nM) or 4OHT (0–1000 nM) (Fig. 4d).^26^ Therefore, the temporal control of CRISPR/Cas9 gene editing can be achieved by administering TMP or 4OHT at specific concentrations and time points.^26,69,86^ These small-molecule inhibitors are especially useful when CRISPR/Cas9 gene-editing activity must be restricted within a narrow temporal window or specific cells.

### Bioresponsive delivery carrier

**Fig. 5:**
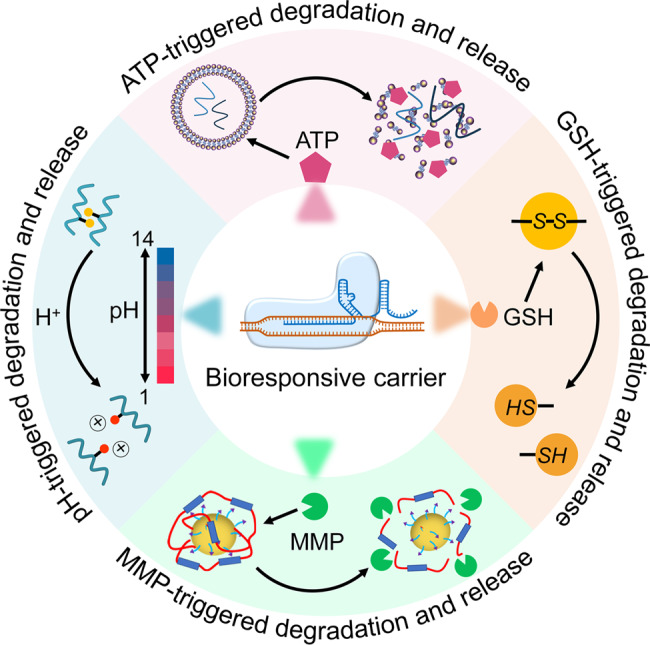
Bioresponsive carriers for controllable release of CRISPR gene editing kits in specific tissues

For CRISPR gene therapy, virus-based carrier and non-viral carrier delivery strategies have been explored to implement the transfer of the therapeutic CRISPR system.^52,87–91^ Adenovirus (Ad), adeno-associated virus (AAV), lentivirus and retrovirus are all gene transfer vectors widely used in gene therapy, and their structures, replication cycle and usage have been thoroughly studied.^92–94^ Research on nonviral carriers, often in the form of nanoparticles, has also been very popular in recent years.^87,95–100^ These CRISPR carriers would be better endowed with some smart functions, such as the stable encapsulation of CRISPR/Cas9 in the transport, biocompatibility, targeting ability, and controlled release of editing tools at the desired site, that are beneficial to overcome biological barriers.^98,101^ Recently, researchers have designed many bioresponsive delivery carriers.^102–111^ The main mechanism by which these bioresponsive delivery carriers achieve the spatiotemporal control of CRISPR/Cas9 gene editing is changing their structures in response to biological signals, such as redox potential, enzymatic activities, ATP and pH in microenvironments at a specific location, and then control the release of payloads in carriers (Fig. 5).

#### Redox-bioresponsive

Redox-bioresponsive delivery carriers enable controllable release of the cargos through disulfide bond cleavage in the reductive cytosolic microenvironments (Fig. 5).^112,113^ For example, Liu et al. designed a bioreducible BAMEA-O16B lipid nanoparticle with disulfide bond-containing hydrophobic tails, which could encapsulate the Cas9 mRNA/sgRNA complex via electrostatic interaction to assemble lipid/Cas9 mRNA/sgRNA nanoparticles for mRNA delivery and CRISPR/Cas9 genome editing.^114^ Once the nanoparticles enter the cell, they release mRNA following reductive chemical signals through a disulfide bond exchange mechanism, resulting in a 90% GFP knockout efficiency in human embryonic kidney cells.^114^ Bioreducible liposome nanoparticles are one of the most mature vectors currently used for CRISPR/Cas9 gene editing. They can promote the application prospects of mRNA therapeutics and the CRISPR/Cas9 technique.

#### pH-bioresponsive

pH-bioresponsive delivery systems can respond to different pH values (Fig. 5).^103^ Tumor tissues, endosomes, and lysosomes have distinct pH characteristics,^102,115^ such as an acidic milieu (pH 4.5–6.5) for endosomes and lysosomes and a slightly acidic environment (pH ~6.5) in solid tumor tissues.^103^ The acidic environment in tumoral tissues and endosomes can trigger the cleavage of covalent bonds, leading to conformational changes in pH-sensitive moieties of the polyplexes (Fig. 5).^116,117^ These pH-sensitive moieties can be used as part of the functionality in a bioresponsive delivery carrier for endosomal escape and release of payload in cancer cells.^118–120^ Imine and ortho ester groups are acid-responsive groups that can be easily degraded in the acidic endosomes of cancer cells. These groups were used by Qi et al. to design a fluorinated acid-responsive polycation (ARP-F).^120^ Negatively charged plasmids, including reporter plasmids pEGFP-N1 and Cas9 plasmid pCas9-surv, can be recruited to the positively charged ARP-F to form a stable ARP-F/pCas9-surv nanoparticle via electrostatic interaction.^120^ Acid-liable ortho ester linkages are relatively stable under neutral pH microenvironments. However, in an acidic or a slightly acidic microenvironment, the bond breaks to release the pCas9-surv plasmids. Thus, when ARP-F/pCas9-surv nanoparticles with acid-liable ortho ester linkages accumulate in lung cancer A549 cells, the slightly acidic pH triggers ortho ester linkage cleavage and releases pCas9-surv, which further encodes the Cas9 protein and sgRNA to knock out the target gene.^120^ This pH-responsive shielding system is a potent strategy for targeted delivery of the genome editing machinery.

#### Enzyme-bioresponsive

Enzyme-bioresponsive delivery carriers often involve some enzymes overexpressed by tumors, such as matrix metalloproteinases (MMPs) which can break down the extracellular matrix of tumor tissue (Fig. 5).^121^ Thus, exploiting this property and introducing MMP-sensitive peptide linkers (e.g., the peptide sequence PLG*LAG) to polyplex carriers, tumor targeting can be achieved because of the breakdown of polyplexes by MMPs of the tumor tissue (Fig. 5), resulting in the promotion of payload internalization into tumor cells.^122,123^ For example, Veiman et al. utilized the cell-penetrating peptide PepFect14 (PF14), which is dual-functionalized with polyethylene glycol (PEG) and a matrix metalloprotease (MMP) substrate, to form nanoparticles. The condensed PF14 and plasmid DNA are sheltered by PEG in an MMP-responsive manner.^122^ When intravenously administered, the complexes exhibit in vivo delivery and efficient expression of pDNA in tumor tissues, avoiding normal tissues.^122^ However, only a few studies have reported on the application of enzyme-responsive carriers in CRISPR gene editing. This strategy can be imitated to deliver CRISPR/Cas9 plasmids, wherein the CRISPR/Cas9 plasmid can be released in tumor tissues to conduct tumor-specific gene editing.

#### Multiple stimuli-bioresponsive

However, using only a single endogenous bioresponsive stimulus to design a spatially targeted CRISPR vector cannot satisfy the pursuit of scientists. Recently, scientists have even tried to develop more versatile bioresponsive delivery platforms based on multiple stimuli for better spatiotemporal control of CRISPR gene editing in target cells or tissues.^124,125^ One of the latest examples is to combine the characteristics of near-infrared (NIR) sensitivity and the endogenous reduction effect to fabricate NIR-sensitive and reducing agent-sensitive nanoparticles.^124^ The nanoparticle comprises following parts: (1) nitrilotriacetic acid-disulfanediyldipropionate-polyethyleneglycol-*b*-polycaprolactone (NTA-SS-PEG-PCL); (2) photosensitizer chlorin e6 (Ce6); (3) cationic copolymer iRGD-PEG-b-polyasparteg-1,4-butanediamine (internalizing RGD-PEG-pAsp(DAB)); and (4) gene editing tool Cas9/sgRNA.^124^ NTA-SS-PEG-PCL can self-assemble into micellar nanoparticles (NTA-NPs), which contain a hydrophobic end (PCL) to encapsulate photosensitizer Ce6, a hydrophilic end (NTA-SS-PEG) to bind to His-tagged Cas9 RNP, and plentiful reducible disulfide linkages in NTA to control the release of Cas9 in the cytoplasm.^124^ NTA-NPs loaded with Ce6 and Cas9/sgRNA are termed CC-NPs, which are then coated with the cationic polymer iRGD-PD to introduce tumor-targeted ligand iGPD and form new complex iGPD-CC-NPs (referred to here as T-CC-NPs).^124^ With the help of tumor-targeted ligand iGPD, T-CC-NPs are accumulated on the tumor cell membranes and are internalized. Under NIR irradiation, Ce6 will generate reactive oxygen species (ROS), triggering the lysosomal escape of T-CC-NPs.^124^ Once the nanoparticles enter the cytoplasm, the high glutathione (reduced form) (GSH) level in the cytoplasm will cause rupture of the disulfide bond between NTA and PEG, allowing the release of Cas9/sgRNA, which then enters the nucleus for subsequent gene editing.^124^ Another similar study designed a lactose-derived CRISPR/Cas9 delivery system by exploiting two internal bioresponsive stimuli: (1) asialoglycoprotein receptor (termed ASGPr), which is overexpressed on the surface of liver cancer cells and can specifically recognize the galactose residue on lactose, and (2) the breakage of disulfide linkages in a reducing environment.^125^ A lactose-derived branched cationic biopolymer (LBP) was created using a facile one-pot ring-opening reaction of amino group-modified lactose (Lac-NH_2_) and triglycidyl isocyanurate (TGIC).^125^ The lactose in LBP contains two residues: glucose residue and galactose residue. Therefore, it can help the delivery system locate the tumor and promote endocytosis through the specific binding of galactose residue and ASGPr. Additionally, LBP has plentiful reducible disulfide linkages to form reduction-responsive degradable cationic vectors. Under a reducing environment, the disulfide linkages will be broken to promote the release of CRISPR/Cas9, which could be guided into the nucleus for subsequent gene editing.^125^ The above strategies combine internal and external stimuli to realize the spatiotemporal control of CRISPR gene editing and reduce off-target effects. For the external stimulus, controlling the irradiation of NIR in the spatial and temporal dimensions determines whether the nanoparticle escapes from lysosomes at a specific time and site. For internal stimuli, the asialoglycoprotein receptor can mediate the specific binding of LBR to tumors, and the reducing environment in the cytoplasm determines the release of Cas9/sgRNA.

In addition to the bioresponsive stimuli mentioned above, some other triggers, such as ROS, ATP, and hypoxia, can be utilized as endogenous stimuli of bioresponsive delivery systems. Comprehensive overviews of bioresponsive polyplexes for nucleic acid delivery can be found in some excellent reviews.^102,103^

Notably, some recent studies reported using of selective organ-targeting (termed SORT) nanoparticles for tissue-specific mRNA delivery and CRISPR/Cas9 gene editing.^126,127^ The authors accurately and predictably optimized traditional lipid nanoparticles (LNPs) to quickly achieve targeted mRNA delivery and CRISPR/Cas9-mediated gene editing in the liver, lung, and spleen tissues.^126^ Generally, the strategy to achieve this goal is to introduce lipid molecules with different proportions of charges (termed SORT molecules), such as cationic lipids (DOTAP, DDAB, and EPC), anionic lipids (18PA, 14PA, and 18BMP) and ionizable cationic lipids (DODAP, C12-200, and 5A2-SC8 molecules), into traditional LNPs (mDLNP, MC3 LNPs, and C12-200 LNPs) to obtain a series of SORT LNPs such as DOTAP mDLNP and 18PA mDLNP.^126,128^ After the intravenous injection of low-dose (0.1 mg/kg) luciferase mRNA (Luc mRNA) LNPs, DOTAP mDLNP and 18PA mDLNP successfully transfected organs in mice. Surprisingly, using different proportions of DOTAP and 18PA, organ-selective mRNA delivery was demonstrated: liver-specific transfection (0% DOTAP), spleen transfection (10–15% DOTAP), and lung-specific transfection (50% of DOTAP).^126^ Introducing 5–40% 18PA, mDLNP delivered mRNA specifically to the spleen, but no luciferase expression was detected in the liver and lung.^126^ After a single intravenous administration of SORT LNPs encapsulating Cas9 mRNA (or Cas9 protein) and sgRNA complexes in tdTom mice, red fluorescent signals were observed in the liver and lungs.^126^ In summary, cationic SORT lipids can control mRNA targeted delivery in the spleen and lung, anionic SORT lipids can achieve mRNA-specific spleen delivery, and ionizable SORT lipids can be used to enhance liver mRNA transfection efficiency. SORT technology can adjust the selectivity of LNPs targeting organs by adjusting the internal charge of LNPs and independent of the type and chemical structure of LNPs. This technology provides a predictable, accurate, and rapid method for screening organ-selective LNPs, and has wide applicability.

### Comparison of different chemical strategies

Chemical strategies using small-molecule activators, small-molecule inhibitors, and bioresponsive delivery carriers provide promising approaches for the spatiotemporal control of CRISPR/Cas9 gene editing in vivo. Nevertheless, each has its limits (Table 4).

To some extent, the use of small-molecule activators and small-molecule inhibitors to control CRISPR/Cas9 gene editing has certain similarities, both of which can directly regulate the activity of the Cas9 protein by controlling the conformational changes of Cas9. As for small-molecule inhibitors, it also can conduct the timely degradation of Cas9 to control CRISPR/Cas9 gene editing. Small-molecule inhibitors or activators have some similar advantages: (1) they are cell-permeable and stable to proteases and may be nonimmunogenic; (2) they exhibit fast kinetics, and allow precise temporal control; and (3) they have good reproducibility.^8,14^ However, identifying or screening Cas9 small-molecule inhibitors or activators remains a challenge. Additionally, most small-molecule activators, particularly rapamycin and doxycycline, may induce potential cytotoxicity and adversely impact the organism’s microbiome. Moreover, small-molecule-activated systems sometimes have intense background activities even in the absence of small-molecule activators, hindering precise temporal control of CRISPR/Cas9 in vivo.^8,18,69,129,130^

The design of bioresponsive delivery carriers by installing bioresponsive moieties into carriers enables the well-programmed multifunctionality of the CRISPR/Cas9 system because of their potential arbitrary modifiability. This strategy can achieve the desired functionality, such as the accumulation and intracellular release of the CRISPR/Cas9 system in target tumors in response to different biological stimuli in the microenvironment. However, this strategy is limited to only a few bioresponsive stimulating factors and remains underutilized in clinical applications. Thus, the design of delivery carriers with diverse and multiple biological stimuli is a further research direction for the precise spatiotemporal control of CRISPR/Cas9 gene editing.

## Physical control

**Fig. 6:**
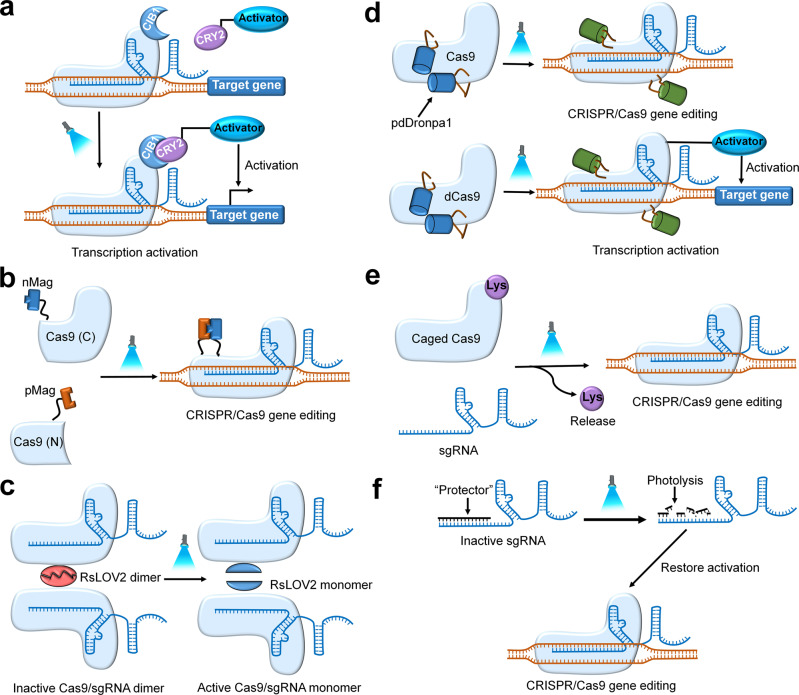
Light strategies to regulate the activity of CRISPR/Cas9 system. **a** A photoactivatable CRISPR/Cas9 transcription system. Blue light illumination causes the formation of dCas9-CIB-CRY2-activator complex which can promote target gene transcription. **b** A photoactivatable split Cas9. Blue light illumination can promote the heterodimerization of the split Cas9 fragments via pMag-nMag interaction, leading to the restoration of Cas9 activity. **c** Cas9-RsLOV2 homodimer dissociates under blue light irradiation, allowing the release of Cas9-RsLOV2 monomer and getting rid of the steric inhibition effect, thus restoring the activity of Cas9. **d** With the illumination of 500 nm light, pdDronpa1 dimer will be dissociated, leading to the reactivation of Cas9 or dCas9 activity. **e** The insertion of photocaged lysine to a specific domain of the Cas9 protein can make Cas9 inactive, until the photocaged lysine is removed under the light irradiation. **f** The photocleavable ssDNA oligonucleotide termed as “protector” can bind to the sgRNA and block CRISPR activity until the protector oligonucleotides are photolyzed by UV irradiation

**Fig. 7:**
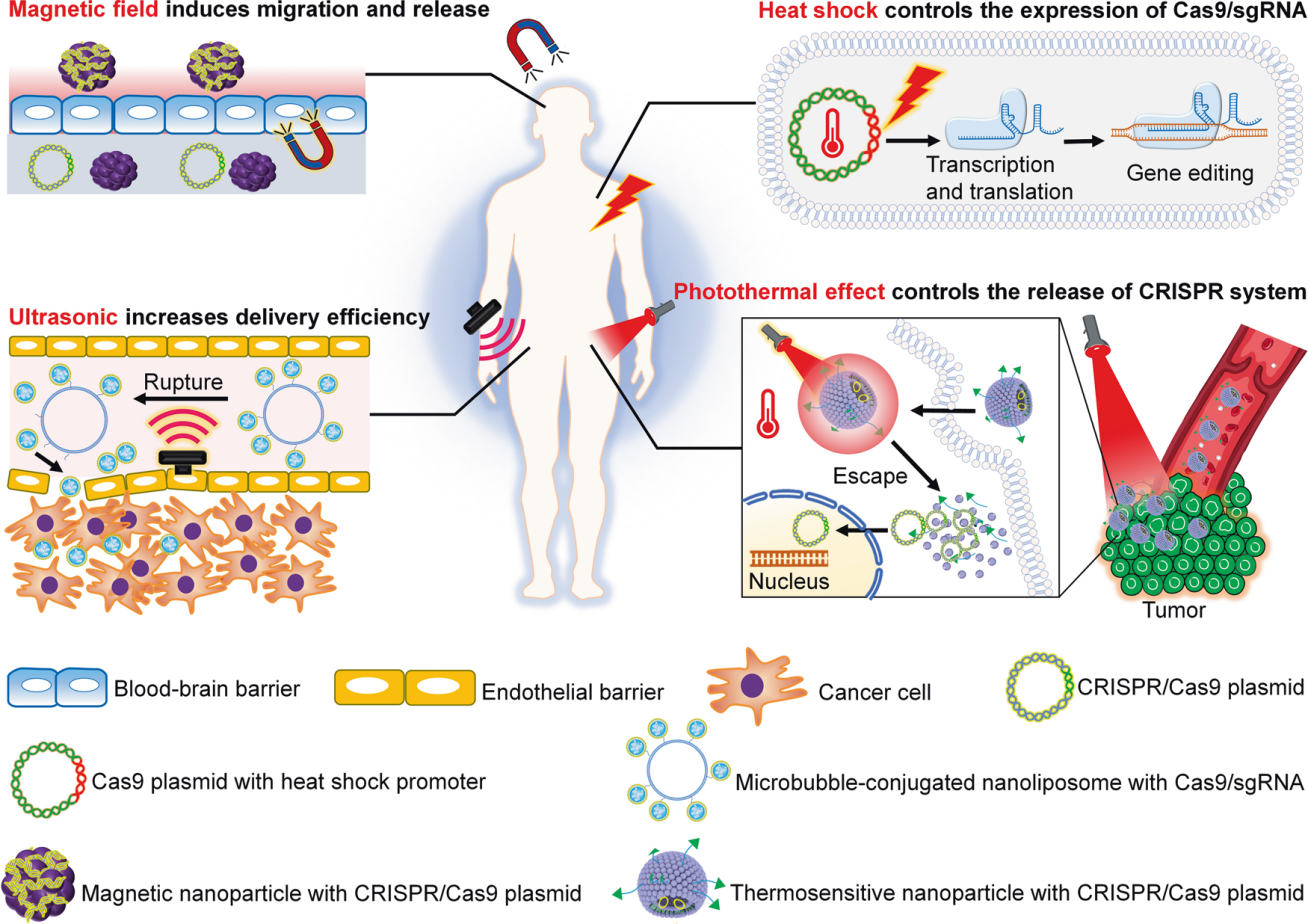
The different physical strategies for remote control of CRISPR gene editing

In recent years, physical control has become an increasingly popular strategy in the spatiotemporal control of the CRISPR/Cas9 gene editing system due to its high spatiotemporal precision and noninvasiveness.^18,131^ Generally, the main mechanism of the physical strategy is that some physically responsive elements, such as optical-responsive, heat-responsive, magnetic- and ultrasound-responsive components, are used to construct CRISPR platforms or delivery carriers (Figs. 6,7). Following the stimulation of different physical factors, the structure, activity, function, expression, transport, and release of the CRISPR/Cas9 system can be controlled in time and space dimensions. In the following sections, four types of physical strategies are discussed, including light control, heat control, ultrasonic control, and magnetic field control (Table 3).

**Table 3:** Examples of physical spatiotemporal control of CRISPR gene editing

Control type	Cell or organism models	Edited gene	Key molecule/structure	Ref.
Light	HEK-293T cell, ZF4 cell, HeLa cell, *E. coli*	zebrafish *ASCL1a* and *HSP70* gene, human *GRIN2B* gene, *CD71* gene, the promoter of the human *ASCL1* and *IL1RN* gene, *mCherry* reporter gene	photocleavable ssDNA oligonucleotide, dimeric fluorescent protein pdDronpa1, CIB1-CYR2-effector, pMag-nMag, Cas9-RsLOV2 monomers, photocaged lysine	^28,136,137,139,140,142,145^
Heat	HCT 116 cell, HEK-293T cell, A375 cell, *C. elegans*	*Plk-l* gene, *GFP* reporter gene, *C. elegans* somatic cell *DPY-5*, *LON-2*, and *GFP* gene	P*hsp*, APC, AuNPs, SPPF-Dex nanoparticles	^18,55,146,147^
Ultrasound	B16F10 cell, DPC cell, androgenic alopecia, mouse	*GFP* reporter gene, steroid type *II 5-alpha-reductase* gene	gold nanowires (AuNWs), microbubble conjugated nanoliposome (MB-NL)	^149,150^
Magnetic field	microglial (hμglia)/HIV (HC69) cell, Hepa 1-6 cell	*HIV LTR* gene, mouse *VEGFR2* gene	magneto-electric nanoparticles (MENPs), magnetic iron oxide nanoparticles (MNP-BV)	^9,36,160^

*HEK-293T cell* human embryonic kidney 293T cell, *ZF4 cell* zebrafish embryonic fibroblast, *DPC cell* dermal papilla cell, *GFP* green fluorescent protein, *CD71* cell surface transmembrane proteins gene, *Plk-l gene* gene for regulator of mitosis, *APC* a cationic polymer-coated Au nanorod, *Phsp* heat-shock-inducible promoter, *AuNPs* lipid encapsulated gold nanoparticles, *SPPF-Dex NPs* consists of alkyl side chains, dexamethasone (Dex), fluorinated polyethylenimine (PF) and PEG chains

### Light

Over the past decade, several photoresponsive molecules have been screened to engineer optically controlled CRISPR gene editing systems.^8,23,35,132,133^ Azobenzene derivatives, spiropyran derivatives, and a group of photosensitive molecules containing o-nitrobenzyl moieties are all photoresponsive molecules that readily undergo photoisomerization or ester bond cleavage under light shock.^134,135^ Exploiting these characteristics, different light-controlled CRISPR/Cas9 gene editing technologies can be designed. Three main strategies exist for light-controlled CRISPR gene-editing technology.

The first strategy is to expand the functionality of the Cas9 protein by light induction. For example, some studies have reported an optogenetic two-hybrid system, which contains two independent components: a genomic anchor (dCas9 system) fused to the light-sensitive cryptochrome-interacting basic-helix-loop-helix (CIB1) protein to form the dCas9-CIB1 complex and a cryptochrome circadian clock 2 (CRY2) fused to a different effector domain (activating effectors) to form the CRY2-activator complex (Fig. 6a).^29,136–138^ Under the stimulation of blue light (peak ~450 nm), the CIB1-effector complex could be recruited to form the biopolymer dCas9-CIB1-CYR2-effector complex, expanding the activation functionality of Cas9 (Fig. 6a).^29,136,138,139^ Moreover, incubating cells in the dark can reverse this activation.^29,138^

The second strategy is to change the Cas9 nuclease activity by light induction. For example, Nihongaki et al. described an engineered photoactivatable split Cas9 (termed paCas9) comprising nitrogen- and carbon-terminal fragments that are fused to light-inducible dimerization domains (pMag and nMag) (Fig. 6b).^140^ Without light stimulation, each split fragment from Cas9 is inactivated. However, blue light illumination can promote the heterodimerization of split Cas9 fragments via pMag-nMag interactions, leading to the restoration of Cas9 activity (Fig. 6b).^140,141^ Furthermore, paCas9 and wild-type Cas9 have similar nuclease activity and targeting specificity. Thus, paCas9 can be utilized in genome editing and genomic modifications, allowing the possibility to conduct spatiotemporal control of CRISPR gene editing via the spatiotemporal control of blue light irradiation. In contrast to paCas9, a different photoswitchable Cas9 was designed (named psCas9) that employs a single-polypeptide architecture (Fig. 6d).^142^ The REC2 and PI domains of psCas9 were inserted by the photodissociable dimeric fluorescent protein (pdDronpa1) (Fig. 6d).^132,143^ Without treatment with 500-nm light, the inserted pdDronpa1 domains homodimerize and subsequently sterically inhibit psCas9 activity. However, following the illumination with 500-nm light, pdDronpa1 dissociates, resulting in the restoration of the Cas9 activity, genome editing functions, and transcriptional upregulation (Fig. 6d).^132,142,143^ A similar study by Richter et al. developed a Cas9-RsLOV2 monomer, which was constructed by the fusion of Cas9 and the *R. sphaeroides* LOV domain (RsLOV) (Fig. 6c).^144^ Without light stimulation, two Cas9-RsLOV2 monomers can homodimerize, causing severe steric inhibition of Cas9 activity. However, under blue light shock, the Cas9-RsLOV2 dimer can dissociate and revert to the Cas9-RsLOV2 monomer, which has high nuclease activity and targeting specificity (Fig. 6c).^132,144^ Another way to change Cas9 activity by light induction is to insert a photocaged lysine to the specific domain of Cas9 that plays an essential role in binding to gRNA, rendering Cas9 inactive (Fig. 6e).^145^ Under exposure to ultraviolet light for 120 s, the photocaged group can be removed, leading to restoration of Cas9 activity and subsequent gene editing or transcriptional upregulation (Fig. 6e).

The third strategy is to change the sgRNA activity by light induction. One of the most typical examples was reported by Jain et al., who designed a photocleavable ssDNA oligonucleotide, termed “protector”, that couples to the target region of sgRNA (Fig. 6f).^28^ Once the photocleavable protector hybridizes to the sgRNA, inhibition of sgRNA:DNA base pairing occurs until the ssDNA oligonucleotide is photolysed by UV irradiation, releasing sgRNA from it to bind target DNA again and undergo subsequent gene editing. However, this method does not have a reversible property.

### Heat

Recently, heat shock or photothermal effects have been used to remotely switch on gene expression (Fig. 7).^146,147^ Depending on the site of heat shock, this strategy can be divided into two categories. One category controls the spatiotemporal release of the CRISPR/Cas9 system via heat shock of carriers. For example, Wang et al. engineered a thermosensitive CRISPR/Cas9 release system using lipid-encapsulated gold nanoparticles (AuNPs).^146^ First, cations and cell nucleus-targeting TAT peptides are linked to AuNPs via sulfhydryl linkage to construct cationic AuNPs. Then, by electrostatic interaction, the negatively charged Cas9-sgPlk-1 plasmid (CP) is condensed on the cationic AuNPs to produce the complex AuNPs/CP (ACP), which is further coated by lipids to form lipid-encapsulated ACP (LACP). Because the localized surface plasmon resonance (LSPR) of AuNPs can generate heat, the AuNPs in the LACP can localize the heat source to trigger TAT/CP release from the AuNPs via photothermal effects under 514-nm laser irradiation. The TAT/CP complex is guided into the cell nucleus by TAT so that the targeted gene (*Plk-1*) is effectively knocked down and tumors are inhibited in vivo.^146^ Similarly, Li et al. designed another photothermal-triggered CRISPR/Cas9 release system using a semiconducting polymer brush (SPPFs), which has NIR-II imaging and photothermal transducer characteristics.^147^ Thus, compared with the LACP system designed by Wang et al.,^146^ the system adds one functionality of NIR-II imaging that can track the distribution of the gene editing tools in the body after administration and to conduct remote photothermal triggers in real time. Briefly, the system comprises three elements: (1) the brush-structure SPPFs, serving as the roles of NIR-II imaging, photothermal transducer and payload carriers; (2) dexamethasone (Dex) wrapped around the core of the formed SPPF nanoparticles to form complexes (SPPF-Dex), which can bind to nuclear glucocorticoid receptors to expand nuclear pores; and (3) CRISPR/Cas9 cassettes that can bind to PF via electrostatic interaction for target gene editing.^147^ After administration, the distribution of SPPF-Dex nanoparticles in the body can be tracked by NIR-II imaging. When reaching the target cell or tissue, the nanoparticles generate heat via the photothermal effect with laser irradiation at a wavelength of 808 nm, facilitating the endolysosomal escape of nanoparticles and release of the CRISPR/Cas9 and Dex payloads from the nanoparticles to the cytosol. With the help of Dex, CRISPR/Cas9 cassettes efficiently enter the nucleus for a series of subsequent biochemical reactions and target gene editing.^147^ The LACP or SPPFs-CRISPR/Cas9 system provides a versatile method for the spatiotemporal control of CRISPR gene editing through spatiotemporal control of the photothermal-trigger.

Heat shock can also spatiotemporally activate the heat shock promoter (P*hsp*) of CRISPR/Cas9 cassettes (Fig. 7), resulting in conditional gene editing in different cell types at different developmental stages.^18,55,148^ For example, Shen et al. reported that the CRISPR/Cas9 plasmid controlled by heat shock successfully produced conditional gene knockout in *C. elegans*.^55^ First, they designed a plasmid expressing Cas9 protein and sgRNA, which were driven by the P*hsp* and U6 promoters, respectively. The plasmids were then injected into the *C. elegans* germline and heated at 33 °C for 1 h to activate P*hsp*, which was switched off by simply cooling the temperature to 20 °C.^55^ Under the control of P*hsp*, the CRISPR/Cas9 system showed robust and time-dependent target gene editing via heat shock. Recently, studies have combined P*hsp* with photothermal carriers,^18^ which seem more convenient to switch the activity of P*hsp* on/off via photothermal effects. For example, Chen et al. designed a nanosystem (termed nanoCRISPR) comprising cationic polymer-coated Au nanorods (APCs), heat-shock promoter (HSP)-driven Cas9 plasmids and heat-shock factors (HSFs).^18^ APC not only acts as a carrier for plasmids delivery but also serves as a local heat source (absorbing the external NIR-II photonic energy and converting it into local heat) to induce conformational change in the HSF monomer to form an HSF trimer, which can activate HSP to promote Cas9 endonuclease gene expression.^18^ Once NIR-II irradiation is switched off, the photothermal effects disappear, resulting in a decrease in the system temperature. The decreased temperature triggers the decomposition of the HSF trimer into the HSF monomer, causing inactivation of the transcription process of the CRISPR/Cas9 plasmid. Thus, gene editing can be easily and precisely controlled by fine-tuning the NIR-II irradiation time at multiple time points in vitro and in vivo.^18^

### Ultrasound

Similar to heat and light control, the application of ultrasound to control the release of payloads from carriers has attracted increasing attention (Fig. 7).^149,150^ The key to using this technology is the carrier design. Many nanomotors that can convert chemical fuels or external energy into mechanical motion have attractive characteristics,^151–153^ including propelled motion and cargo transportation, enabling them to be implemented in biosensing and drug delivery.^154–156^ In particular, ultrasound-driven nanomotors have been shown to rapidly penetrate cell membranes and maintain acoustic activity in the intracellular space, making them good vehicles to conduct intracellular drugs delivery (Fig. 7).^157^ Recently, Hansen-Bruhn et al. reported using ultrasound-propelled nanomotors as carriers to directly and quickly deliver functional Cas9/sgRNA complexes into cells under ultrasound activation.^149^ The Cas9/sgRNA expression plasmid is connected to the surface of gold nanowires (AuNWs) via disulfide bonds to form the Cas9/sgRNA@AuNW complexs, which can generate active movement under ultrasound activation to promote their internalization into the cytoplasm. Microbubble-conjugated nanoliposomes (MB-NLs) are another material that can be applied as carriers for Cas9/sgRNA riboprotein complexs. MB-NL can significantly promote local delivery to target sites under ultrasound activation (Fig. 7).^150^ Ryu et al. designed an ultrasound-activated microbubble-conjugated nanoliposome (MB-NL) system to deliver Cas9/sgRNA and conduct androgenic alopecia therapy.^150^ MB cavitation-induced sonoporation of the carrier particle can boost the delivery efficiency of the Cas9/sgRNA complexes to the dermal papilla cell (DPC) under high acoustical wave ultrasound frequency (1–5 MHz).^150^ The MB-NL and Cas9/sgRNA@AuNW systems improve the local delivery of gene editing tools via ultrasound stimulation, with unique advantages such as safe transportation at specific sites.

### Magnetic field

Previous studies have shown that magnetic nanomaterials can change molecule or cellular behaviors both in vitro and in vivo under the stimulation of an external magnetic field.^158,159^ Therefore, these magnetic nanomaterials may also be used to construct CRISPR/Cas9 system carriers for noninvasive delivery and on-demand release to target tissues or cells (Fig. 7).^9,160^ Kaushik et al. reported magnetically guided noninvasive delivery of a nanoformulation (NF) containing a Cas9/gRNA gene editing system bound with magneto-electric nanoparticles (MENPs) (Fig. 7).^160^ MENP-Cas9/gRNA nanomaterials driven by a magnetic field can cross the blood-brain barrier (BBB) to edit the HIV-1 gene and reduce latent HIV-1 infection in microglial (hμglia)/HIV (HC69) cells.^160^ The carriers (MENPs) have the characteristics of being ferromagnetic, nontoxic (up to 50 µg), and 25 ± 5 nm in size, allowing them to pass through the BBB under a static magnetic field; at the same time, MENPs can cause polarization changes on their surface when stimulated by an external ac-magnetic field, resulting in bond breakdown between Cas9/gRNA and MENPs and benefiting Cas9/gRNA on-demand release in target tissues to conduct subsequent gene knockout or gene mutation.^160^ In another study, Zhu et al. reported recombinant magnetic nanoparticles baculoviral vectors (MNP-BV-CRISPR) could mediate CRISPR/Cas9 gene editing at a specific site via a magnetic field.^9^ MNP-BV-CRISPR nanoparticles can disperse in aqueous buffers and migrate against the field gradient as nanomagnets following exposure to a magnetic field.^9^ Additionally, MNP-BV-CRISPR can be inactivated by the complement system in serum, thus serving as an “off” switch of gene editing, and an external magnetic field can be used as an “on” switch by locally controlling the margination and cell entry of the MNP-BV-CRISPR nanoparticles to conduct gene specific editing.^9^ MENPs-Cas9/gRNA and MNP-BV-CRISPR show the possibility of using magnetic stimulation to conduct spatiotemporal regulation of CRISPR gene editing in vivo. However, the application of this spatiotemporal regulation in vivo has not been fully studied.

### Comparison of different physical strategies

Each of the four different physical approaches for the spatiotemporal control of CRISPR gene editing has disadvantages and advantages (Table 4). Light control has become an increasingly popular tool in the spatiotemporal application of CRISPR gene editing. Its main mechanism is to insert some photoresponsive molecules, which can readily undergo photoisomerization or ester bond cleavage under light shock, into Cas9 or sgRNA structures,^134,135^ resulting in spatiotemporal control of the functionality or activity of Cas9 and sgRNA. Light regulation often has the advantages of low toxicity, reversible properties, high spatiotemporal precision, and noninvasiveness,^148^ but shortcomings exist:^69^ (1) the intense absorption and scattering of light by turbid tissues; (2) the requirements of long-term exposure (tens of minutes to several hours) and high light intensities; and (3) the limitation of light resources to a narrow spectral range, preventing them from in vivo applications.

**Table 4:** Advantage and disadvantage for different strategies

Control type	Advantage	Disadvantage	Ref.
Small molecule activators	(1) Easy to synthesis (2) Cell permeable and nonimmunogenic (3) High level of gene editing efficiency	(1) Potential cytotoxicity (2) Significant background activity (3) Lack wide-dynamic range	^8,14,18,69^
Small molecule inhibitors	(1) Easy to synthesis (2) Cell permeable and nonimmunogenic (3) The low level of off-target efficiency	(1) Significant background activity (2) Lack of easy tunability in clinical application (3) Difficulties in identification and screening	^8,14,26,27,69^
Cell-specific promoters	(1) The low level of off-target efficiency (2) High gene editing specificity in the spatial dimension	(1) Difficulties in screening of more promoter with high cell specificity and high activity	^37,57^
Bioresponsive delivery carriers	(1) High capacity (2) With programmed functionalities (3) High gene editing specificity in the spatial dimension	(1) Limited by endogenous stimuli (2) Difficulties in screening of bioresponsive moieties and advanced materials used to synthesize carriers	^114,119,120,122,126^
Light control	(1) Noninvasiveness (2) Low toxicity (3) Reversible property (4) Easy tunability	(1) Long exposure time (2) Limited to a narrow spectral range (3) Strong absorption of light by turbid biological tissues	^69,126,134,136,137,139–141^
Heat control	(1) Noninvasiveness (2) Easy tunability (3) High gene editing spatiotemporal specificity	(1) Long exposure time (2) Low photothermal conversion efficiency (3) Difficulties in screening of more heat shock promoters	^18,55,146,147^
Ultrasound control	(1) Noninvasiveness (2) High transfer efficiency (3) High gene editing spatiotemporal specificity	(1) Less reversible (2) Not been fully explored and underutilized (3) Lack of easy tunability in clinical application	^149,150^
Magnetic control	(1) Noninvasiveness (2) Not disturbed by tissues (3) Low cytotoxicity (4) High gene editing spatiotemporal specificity	(1) Not been fully explored and underutilized (2) Difficulties in screening of magnetic sensing materials	^9,160^

Compared with light methods, the mechanism of heat control is to achieve spatiotemporal CRISPR gene editing by mainly controlling the release or expression of the CRISPR system from designed CRISPR toolkits at specific sites and times.^55,146,147^ Similar to light control, heat control has a high level of spatial specificity, however, the relatively long-term heat shock response and low efficiency of heat transmission hinder their wide applications in vivo. There are two key points in the application of heat control: one is to explore additional heat-responsive delivery carriers with the characteristic of high photothermal conversion efficiency because the heat sources are often obtained from the photothermal effect of the designed carriers under laser irradiation; the other is to discover additional heat shock promoters with high specificity and activity for target tissues or cells.

Ultrasonic control and magnetic control are also classic methods for the noninvasive transportation of the CRISPR gene editing system to target tissues or cells. Both aim to improve the efficiency of Cas9 system delivery and release at a specific site and have spatiotemporal specificity.^149,150,160^ Compared with light and heat stimuli, the magnetic field and ultrasound are not significantly weakened by the tissue and the low-intensity magnetic field and low-frequency ultrasound have no obvious side effects on the human body.^149,150,161^ However, ultrasonic regulation and magnetic regulation are less reversible. The use of magnetic fields or ultrasound to control the delivery and release of the CRISPR/Cas9 system from carriers in vivo or in vitro to achieve spatiotemporal control of CRISPR gene editing has not been fully explored and is underutilized. Diverse materials responsive to magnetic fields or ultrasound should be further explored, providing new ideas for the design of magnetic or ultrasound-responsive delivery carriers. Overall, light, heat, magnetic, and ultrasound can all be used to remotely switch on gene expression. However, light seems to be superior to the other triggers because of its relatively easy operation, versatility, reversibility, and high spatiotemporal resolution.

## Comparison of genetic regulation, chemical, and physical strategies

### Spatiotemporal control

The lack of temporal and spatial precision in the editing process can severely constrain the application of the CRISPR/Cas9 system in complex biological systems. Therefore, gene editing tools with a precise spatiotemporal control ability that can be rapidly and reversibly programmed to target the desired loci at a specific time are of high demand. Genetic regulation, chemical and physical strategies are based on different mechanisms to achieve spatiotemporal regulation of CRISPR/Cas9 gene editing. The mechanism of genetic regulation strategies is to control the editing or transcription of target genes by fusing transcription effectors or constructing cell-specific promoters. For example, the addition of multiple copies of the activation domain VP16 (such as dCas9-VP48, -VP96, and -VP192) improves the transcription activation efficiency of the target gene (Fig. 2b); the specific promoter enables the expression of Cas9 protein only in target cells but not in other cells (Fig. 2g). Chemical strategies mainly control the start, intensity, duration, and site of CRISPR gene editing by adding some small-molecule activators, inhibitors or using bioresponsive delivery carriers. For example, the insertion of inteins into Cas9 leads to Cas9 nuclease inactivation, which can be restored by adding 4-HT (Fig. 3a); the fusion of estrogen receptor (ERT2) squeezes Cas9 out of the cell nuclei (Fig. 3b); the addition of anti-CRISPR protein prevents Cas9/sgRNA from binding to target DNA (Fig. 4a); the destabilized domains DHFR (or ER50) promotes Cas9 proteasomal degradation (Fig. 4c); and selective organ targeting nanoparticles (SORT) rapidly achieves targeted delivery of mRNA and CRISPR/Cas9-mediated gene editing in the liver, lung and spleen tissues by simply adjusting the internal charge. To some extent, small-molecule activators or inhibitors can modulate CRISPR gene editing in the temporal dimension by controlling the point of adding time to decide the onset, intensity, and duration of CRISPR gene editing, but the control in spatial dimension is weaker because of the nonspecific distribution of small molecules in the tissue. By contrast, cell-specific promoter and bioresponsive delivery carriers can achieve better control of CRISPR gene editing in the spatial dimension.

Unlike genetic regulation and chemical strategies, physical strategies achieve spatiotemporal control of CRISPR/Cas9 gene editing by constructing CRISPR platforms or delivery carriers that are photo-responsive, temperature responsive, magnetic field responsive, or ultrasound responsive (Figs. 6, 7). For example, the pdDronpa1 domain modulates Cas9 protein activity via the spatiotemporal control of light irradiation (Fig. 6d); the heat shock promoter (P*hsp*) of CRISPR/Cas9 cassettes allows conditional gene editing in target cells at different developmental stages through a heat shock response; sonoactive gold nanowires (AuNWs) promote Cas9/sgRNA internalization into the cytoplasm by ultrasound activation; MENPs were induced polarization changes on the surface when stimulated by an external ac-magnetic field, resulting in on-demand release of Cas9/gRNA in target tissues. These physical strategies can remotely switch on/off CRISPR gene editing in real-time, enabling easy tunability, noninvasiveness, and high spatiotemporal specificity compared with chemical strategies.

### The off-target effect

Previous studies have mainly focused on improving gene editing efficiency while ignoring off-target effects.^147^ These potential off-target effects in gene editing therapy may lead to undesired results, such as cancer. Therefore, the off-target effect is an important indicator to evaluate the applicability of these strategies.^162^ For genetic regulation strategies, the construction of cell-specific promoter vectors can ensure that Cas9 protein is only translated in target cells, reducing CRISPR editing in nontarget cells and off-target effects (Fig. 2g). Many chemical strategies can diminish the off-target effects of CRISPR gene editing by strictly controlling the insertion site or duration of chemically inducible factors.^27,63,69^ For example, depending on the insertion site of inteins, the ratio of on-target to off-target for both intein-Cas9 variants (intein-Cas9 S219 or C574) was significantly higher than that of wild-type Cas9 (Fig. 3a).^27^ Maji et al. observed enhanced specificity for on-target versus off-target sites of the *VEGFA* gene after treatment with different doses of TMP or 4OHT for the Cas9-DHFR or Cas9-ER50 systems, respectively (Fig. 4c, d).^26^

The easy tunability and high spatiotemporal resolution of physical strategies allow switching on or off CRISPR gene editing activity in real-time, reducing the off-target effect caused by nonspecific accumulation in tissues. Chen et al. recently reported the optogenetically activatable nanoCRISPR that minimizes off-target effects via the optogenetic control of Cas9 expression to avoid prolonged Cas9 activity.^18^ Although various strategies for the spatiotemporal control of CRISPR/Cas9 gene editing have been developed, the possibility of their off-target effects in vivo has not yet been widely studied, and it remains unclear which strategy is the most promising method to reduce off-target effects in different contexts. However, stronger spatiotemporal control ability and specificity was more likely to reduce off-target effects in CRISPR/Cas9 gene editing.

### Critical barriers to clinical translation

The close collaboration between biological scientists and biomaterial researchers has greatly advanced the development of novel strategies for the spatiotemporal control of CRISPR/Cas9 gene editing in vivo and in vitro. However, some problems persist, limiting their extensive application in clinical practice. For example, in gene regulation strategies, the existing cell-specific promoters cannot be readily generalized; thus, screening more effective promoters with cell specificity and high activity is required for this concern. For chemical strategies, several critical barriers exist for clinical translation. First, some chemical methods lack tunability; for example, small-molecule control strategies often require additional factors to be introduced into the target cells, making the process cumbersome to implement in clinical translation. In another method, a split Cas9 architecture is divided into two fragments and subsequently required to restore activity using rapamycin-binding dimerization domains, which may be inconvenient to implement during clinical translation because of the need to operate multiple Cas9 fragments.^63^ Second, the unexpected toxicity associated with using chemicals such as rapamycin and doxycycline may also affect the clinical application. Third, the obvious background activity of some small-molecule-activated systems hinders precise spatiotemporal control of CRISPR gene editing, making it impossible for clinical applications.^8,18,69,129,130^

Similarly, there are also some critical barriers in physical strategies regarding clinical translation. First, the strong absorption of light by turbid biological tissues makes it difficult for optical penetration into deep tissues and some light-mediated activatable CRISPR/Cas9 systems are potentially phototoxic, preventing them from being implemented in clinical applications. Second, the heat source, in many cases, is often obtained from the photothermal effect of the designed carriers. Therefore, the low photothermal conversion efficiency of the carrier can hinder its heat activation. Additionally, heat shock promoters with high activity for target tissues or cells are also insufficient. Third, ultrasonic/magnetic sensing materials that can overcome the depth limit of optical strategies seem to be more accessible to clinical settings and have not yet been fully explored. Fourth, physical strategies often involve knocking signal-responsive components into the CRISPR editing system via synthetic biology to trigger a cascade response, which partially complicates their clinical implementation. Although various strategies have been reported for the spatiotemporal control of CRISPR/Cas9 gene editing, precise regulation in a programmable, inducible manner and with low off-target activity has not yet been demonstrated in clinical applications.

## Conclusion and outlook

The CRISPR/Cas9 system offers unprecedented opportunities to treat various genetic and infectious diseases because of its simplicity and versatility.^163,164^ However, the lack of temporal and spatial precision in the editing process severely constrains the application of the CRISPR/Cas9 system in complex biological tissues. Thus, scientists have exerted much effort to develop CRISPR systems with spatiotemporal control ability using genetic regulation, chemical or physical strategies to reduce undesired genomic targeting, promote dynamic perturbations in the dimensions of time and space, and enable many new opportunities for research on basic theory and application transformation. In this review, we comprehensively summarize the state-of-the-art strategies concerning the spatiotemporal control of CRISPR gene editing, involving conditional expression, light/heat/ultrasound/magnetic activation, bioresponsive delivery, chemical induction, and molecule sequestration of Cas9.

Although these strategies already show some promising preclinical results, still some concerns must be addressed in the future: (1) Packaging capacity: the packaging capacity of the virus is limited, approximately 5 kb, indicating little choice in the size of the sequence that can be packaged into the viral vector.^2,22^ In this regard, researchers use small Cas9 orthologues or dual AAV vectors to partially overcome this problem^165,166^ or design nonviral vectors with high packaging capacity to deliver the CRISPR/Cas9 system. (2) Specificity: CRISPR/Cas9 systems delivered by nonviral vectors can work in nontarget cells, resulting in potential nonspecific gene editing in vivo. After systemic administration, the CRISPR system based on non-viral vectors may also be endocytosed by neutrophils, monocytes, macrophages, and some other phagocytes to accumulate in nontargeted tissues,^167–169^ such as lung, spleen, liver, kidney,^170^ or even after reaching the target tissue, it may also be internalized by nontargeted cells because of the inability to distinguish between diseased cells and healthy cells.^171^ Additionally, scientists have found that after systemic administration, nonviral nanoparticles tend to accumulate in the liver, which not only affects delivery to other target organs but also damages liver tissue, thereby affecting clinical application.^172^ (3) Efficiency: many diseases cannot be effectively cured only by knocking out or mutating the target gene. By contrast, they require modification of the mutant gene or introduction of the desired gene sequence. In this case, HDR is needed to solve this problem. However, during gene editing, the frequency of NHEJ in cells is higher than that of HDR.^172^ Therefore, designing editing tools to increase the frequency of HDR is very important and challenging. (4) Potential immunogenic response: researchers have identified anti-Cas9 antibodies in the human body; thus the CRISPR system may cause an immune response after systemic administration.^173^ Additionally, both viral and nonviral vectors will be recognized by the host immune system in the body and cause an immune response, which can not only seriously reduce the efficiency of gene editing in the body but also threaten the health of the patient.^2^ Fortunately, compared with viral vectors, encapsulating the CRISPR system components in nanocarriers can partially evade the recognition of the host’s immune system, thereby reducing the immune response. (5) Off-target: many reports have indicated that CRISPR gene editing with viruses as vectors may generate the durative expression of CRISPR components, which may severely increase the possibility of genotoxicity and off-target effects in vivo after viral delivery.^95^ For nonviral-mediated genome editing, if it cannot precisely target specific tissues or cells, off-target effects persist. In summary, the efficient and safe delivery of CRISPR/Cas systems into targeted tissues and targeted cell types in vivo to reduce unwanted off-target effects remains a critical challenge for their clinical applications.

For future research, (1) innovative CRISPR system delivery tools that can distinguish diseased cells from healthy cells in clinical trials to reduce off-target effects are needed. Research on intelligent or programmable CRISPR system delivery vehicles that can stay “inert” until being recognized by specific biomarkers, such as enzymes and pH in the microenvironment of disease cells to stimulate the release of the CRISPR system, is expected to solve this problem. (2) The development of nanocarriers that can respond to multiple stimuli in target cells is another direction to explore. Compared with nanocarriers that respond to a single stimulus, multiple-stimuli-bioresponsive nanocarriers can achieve more precise and effective gene editing in clinical applications. In summary, bioresponsive carrier-based CRISPR/Cas9 delivery may be promising for translational applications because of its good versatility and programmable bioactivity. (3) The commonly investigated CRISPR/Cas systems to treat gene diseases are mainly based on nucleases such as Cas9, Cas12a, Cas13a, and their orthologues. Among them, only the CRISPR/Cas systems based on Cas13a can be used to specifically target and cleave RNA. However, very few studies are available on the spatiotemporal control of RNA-targeting CRISPR-Cas13a systems. Discovering switch proteins that can switch on or off CRISPR-Cas13a systems to target RNA is a new direction for future research. (4) Finally, extensive efficacy validation of these strategies in vivo is necessary before clinical applications because most of them have only been tested in cultured cell lines.

## Supplementary information

Editing Certificate
