# Supplementary material for: Spatiotemporal control of CRISPR/Cas9 gene editing
Source: Signal Transduct Target Ther. 2021 Jun 20;6:238. doi: 10.1038/s41392-021-00645-w (PMC8214627; doi:10.1038/s41392-021-00645-w)
Supplement: Supplementary file 1 — Editing Certificate [file 41392_2021_645_MOESM1_ESM.pdf]

SPRINGER NATURE
Author Services

Editing Certificate

This document certifies that the manuscript

Spatiotemporal Control of CRISPR/Cas9 Gene Editing

prepared by the authors

Chenya Zhuo1, Jiabin Zhang1, Jung-Hwan Lee2, Ju Jiao3, Du Cheng4, Li Liu5, Hae-Won Kim2,*, Yu Tao1,*, and Mingqiang Li1,6*

was edited for proper English language, grammar, punctuation, spelling, and overall style by one or more of the highly qualified native English speaking editors at SNAS.

This certificate was issued on **February 9, 2021** and may be verified on the SNAS website using the verification code **8869-722E-6FE1-A465-CB22** .

Neither the research content nor the authors' intentions were altered in any way during the editing process. Documents receiving this certification should be English-ready for publication; however, the author has the ability to accept or reject our suggestions and changes. To verify the final SNAS edited version, please visit our verification page at secure.authorservices.springernature.com/certificate/verify.
If you have any questions or concerns about this edited document, please contact SNAS at support@as.springernature.com.

SNAS provides a range of editing, translation, and manuscript services for researchers and publishers around the world.
For more information about our company, services, and partner discounts, please visit authorservices.springernature.com.
